# Effects of Low-Protein Diets Supplemented with Ketoacid on Expression of TGF-*β* and Its Receptors in Diabetic Rats

**DOI:** 10.1155/2015/873519

**Published:** 2015-12-15

**Authors:** Xiu Yang, Ming Yang, Ming Cheng, Li-Bin Ma, Xiang-Cheng Xie, Shuai Han, Bo Zhang, Xiao Fei, Ming Wang, Chang-Lin Mei

**Affiliations:** ^1^Department of Nephrology, Hangzhou First People's Hospital, Zhejiang 310006, China; ^2^Department of Nephrology, Changzheng Hospital, Second Military Medical University and the Kidney Institute of CPLA, Shanghai 200003, China; ^3^Department of Osteology, Changzheng Hospital, Second Military Medical University, Shanghai 200003, China

## Abstract

TGF-*β*_1_ has been recognized as a key mediator in DN. This study aimed to observe the effects of low-protein diets supplemented with ketoacid on mRNA and protein expression of TGF-*β* and T*β*RI and t T*β*RII receptors in the renal tissue of diabetic rats. A diabetes model was established in 72 male SD rats. They were then equally randomized to three groups: NPD group, LPD group, and LPD + KA group. Additional 24 male SD rats receiving normal protein diets were used as the control. Eight rats from each group were sacrificed at weeks 4, 8, and 12 after treatment, from which SCr, BUN, serum albumin, and 24 h urinary protein excretion were collected. The expressions of TGF-*β*_1_, T*β*RI, and T*β*RII in LPD and LPD + KA groups were significantly lower than those in NPD group and lower in LPD + KA group than those in LPD group. Low-protein diets supplemented with ketoacid have been demonstrated to provide a protective effect on the renal function as represented by reduced SCr, BUN, and urinary protein excretion, probably through downregulating the gene expression of TGF-*β*_1_ and its receptors in LPD + KA group.

## 1. Introduction

Diabetic nephropathy (DN) is the most common cause of end-stage renal disease (ESRD) and one of the leading causes of death in diabetic patients. Despite long-term exploration and application of the clinical therapeutic strategies including control glucose and blood pressure and the use of renin-angiotensin system (RAS) antagonists [1] and lipid-lowering therapies [2], the outcome remains unsatisfactory [3]. It is therefore necessary to explore more effective treatments for DN.

Nutritional therapies as represented by limiting dietary protein intake have entered the field of people's vision [4]. Several studies [5] have demonstrated that low-protein diets (LPD) could protect the renal function and structure in type II diabetes mellitus (DM2) animal models and attenuate macroalbuminuria [6], improve the prognosis [7], and relieve the symptoms [8] in DM2 patients. However, the risk of altered nutrition due to low-protein diets has increasingly become a concern. Ketoacid, a nitrogen-free ketoanalog, has been prescribed together with low-protein diets to treat patients with chronic kidney disease (CKD) [9, 10]. Ketoacids can provide sufficient amounts of essential amino acids and reduce endogenous urea formation, toxic ions, and metabolic products. Animal studies [11] showed that LPD could induce hypoproteinemia and growth retardation in CKD mice, while supplementation with ketoacids could correct these abnormalities. In addition, clinical trials suggested that dietary protein in combination with essential amino acid and ketoanalog supplements could delay the onset of end-stage renal failure without deteriorating the nutritional status of DN patients [12, 13]. However, the mechanism underlying this phenomenon remains unclear.

Advanced glycation end products (AGEs), intracellular polyols, angiotensin II, growth factors, and inflammatory cytokines are believed to play important roles in the occurrence of DN [14, 15]. Of them, TGF-*β*_1_ is a key mediator in DN [16]. In vitro studies have demonstrated that high glucose and AGE can trigger the expression of TGF-*β*_1_ and its receptors in tubular and mesangial cells [17, 18]. It was also found that the expression of renal TGF-*β*_1_ and its receptors was increased in both diabetic patients and animal models [19]. In addition, the increased urinary TGF-*β* level was correlated with the severity of interstitial fibrosis in DN patients, suggesting that there is a link between TGF-*β* expression and progressive diabetic kidney disease [20].

It is well established that LPD supplemented with ketoacids can safely and effectively attenuate renal damage and retard the deterioration of renal function in CKD patients [9, 10]. However, there is no evidence to ascertain the effect of LPD supplemented with ketoacids on the expression of renal TGF-*β*_1_ or its receptors in DN patients. The aim of the present study was to investigate the effect of LPD supplemented with ketoacids on the expression of TGF-*β*_1_ and its receptors in streptozotocin- (STZ-) induced diabetic rats.

## 2. Methods

### 2.1. Animals and Experimental Design

Ninety-six male Sprague-Dawley (SD) rats aged 12 weeks and weighing 280–300 g (Zhejiang Medical Laboratory Animal Center, Hangzhou, China) were housed at optimal temperature with a 12 h light-dark cycle with free access to water and then randomized to a diabetic group (*n* = 72) and a control group (*n* = 24). The diabetes model was established by intravenously injecting STZ (dissolved in citrate, pH = 4.0, Sigma, St. Louis, USA) in a single dose of 50 mg/Kg. Animals that had random blood glucose levels over 16.7 mmol/L in three different times were selected for experiments.

One week after modeling, the 72 diabetic rats were further equally randomized into three diet groups: normal protein diet (NPD) group using 18.5% casein protein, LPD group using 6% casein protein, and LPD supplemented with ketoacid (LPD + KA) group using 5% casein protein and 1% ketoacid. The animal chow (Qualifying Certificate number SCXK (BJ) 2006-0003) was purchased from the Institute of Laboratory Animals of the Chinese Academy of Sciences (Beijing, China).

24-h urine samples were collected from the animals that had been starved for 12 h in the metabolism cage one day before the experiment day. At weeks 4, 8, and 12, eight randomly selected animals from each group were sacrificed to collect the blood and kidneys. The experimental protocol was approved by the ethnic committee of the Second Military Medical University (Shanghai, China).

### 2.2. Determination of Biochemical Parameters

Serum concentrations of albumin (ALB), blood urea nitrogen (BUN), and serum creatinine (SCr) were measured by corresponding commercial kits on an automatic biochemical machine (Toshiba 7170, Japan). 24-h urine samples were collected and quantitated using the standard sulfosalicylic acid method.

### 2.3. Real-Time PCR

Total RNA was extracted from the renal tissue using Trizol reagent (Invitrogen, Carlsbad, CA, USA) according to the manufacturer's protocol, and complementary DNA (cDNA) was synthesized using the Superscript First-Strand Synthesis System. Real-time PCR was performed on ABI 7500 Real-Time PCR System with a SYBR Green real-time PCR master mix kit.

Glyceraldehyde 3-phosphate dehydrogenase (GAPDH) was used as an internal control. The PCR primers used were as follows: TGF-*β*_1_ sense: 5′-AGC CTG CTT CTT GAG TC-3′, antisense: 5′-GAT AATCCG ACA CCA ACC AC-3′; T*β*RI sense: 5′-TCA CTA GAT CGC CCT TTC AT-3′, antisense: 5′-GAT AATCCG ACA CCA ACC AC-3′; T*β*RII sense: 5′-GCG TGG CCG TGT GGA GGA AGA A-3′, antisense: 5′-GGG CAG CAG TTC CGT ATT-3′.

### 2.4. Western Blot Analysis of Protein Expression

The renal tissue was homogenized on ice in modified RIPA Lysis Buffer containing 25 mM Tris-HCl, pH 7.4, 150 mM NaCl, 1 mM EDTA, 1% Tergitol NP-40, 0.1% sodium dodecyl sulfate, 1 mM phenylmethylsulfonyl fluoride, and Protease Inhibitor Cocktail (Sigma-Aldrich, St. Louis, MO, USA). The protein concentration in the tissue homogenate was determined by BSA assay kit (Pierce, Rockford, IL, USA) and 60 *μ*g total protein from each sample was fractionated on 4–12% Bis-Tris gradient gel (Invitrogen, Carlsbad, CA, USA) at 120 V for 2 h and transferred to a nitrocellulose membrane. The membrane was then incubated with rabbit anti-TGF-*β*I or rabbit anti-T*β*RI and mouse anti-T*β*RII antibodies (Invitrogen) at 1 : 250 dilutions and antiactin antibodies (Sigma-Aldrich) at 1 : 10,000 dilutions overnight.

The appropriate horseradish peroxidase-conjugated secondary antibodies (Sigma-Aldrich) were used at a 1 : 5,000 dilution. The membrane was visualized with SuperSignal West Pico (Pierce) and developed by autoluminography.

### 2.5. Statistical Analysis

All measurement data were treated using SPSS 19.0. Measurement data are expressed as the mean ± SD. Differences were determined using two-way analysis of variance (ANOVA) followed by the Student-Newman-Keuls test. *P* values less than 0.05 were considered statistically significant.

## 3. Results

### 3.1. Body Weight, Urinary Protein, and Biochemical Parameters

**Figure 1:**
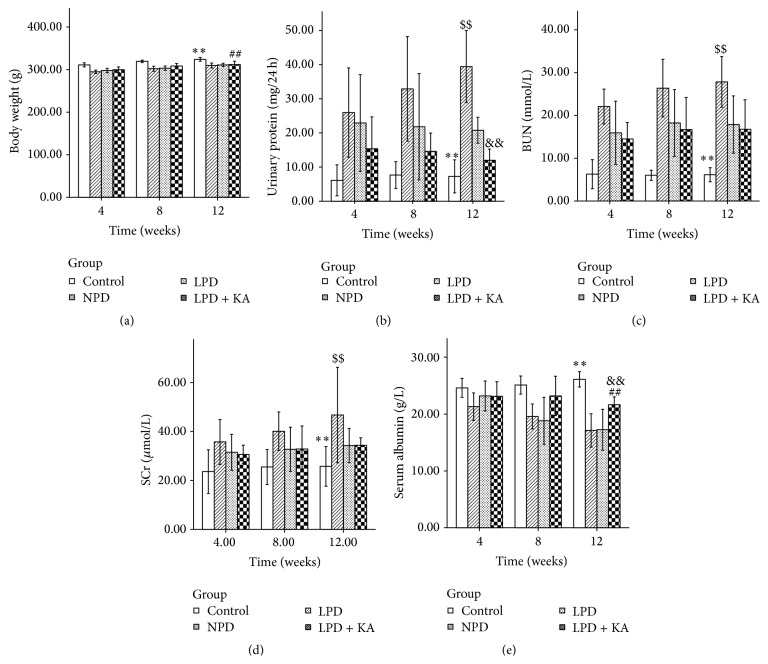
Body weight, urinary protein, and biochemical parameters in the experimental groups. Body weight (a), urinary protein (b), BUN (c), SCr (d), and serum albumin levels (e) in normal rats and diabetic rats fed with NPD, LPD, or LPD + KA. Data are expressed as mean ± SD; ^*∗∗*^ *P* < 0.05 versus NPD, LPD, and LPD + KA; ^##^ *P* < 0.01 versus NPD; ^$$^ *P* < 0.01 versus LPD and LPD + KA; ^&&^ *P* < 0.01 versus LPD. BUN: blood urea nitrogen; SCr: serum creatinine; NPD: normal protein diet; LPD: low-protein diet; LPD + KA: low-protein diet supplemented with ketoacids.

Body weight of all experimental rats increased in varying degrees during the 12-week experimental period. Diabetic rats exhibited remarkably lower body weight than normal rats during the whole experiment (*P* < 0.001). There was no difference between NPD and LPD groups. The mean body weight in LPD + KA group was higher than that in NPD group (*P* = 0.004), probably due to the regulatory effect of ketoacid (Figure 1(a)).

The urinary protein levels in diabetic groups were significantly higher than those in the control group (compared with NPD, *P* < 0.001; compared with LPD, *P* < 0.001; compared with LPD + KA, *P* = 0.017). Urinary protein levels were reduced in rats fed with low-protein diets either with or without addition of ketoacid (*P* < 0.001) as compared with NPD group, but the reduction was more evident in rats fed with low-protein diets with addition of ketoacid (*P* = 0.007) (Figure 1(b)).

BUN and SCr, two parameters reflecting renal clearance function, were decreased after protein restriction (*P* < 0.001) (Figures 1(c) and 1(d)). The serum albumin level of the diabetic rats was lower than that of the normal rats (compared with NPD, *P* < 0.001; compared with LPD, *P* < 0.001; compared with LPD + KA, *P* = 0.002). In addition, the low-protein diet supplemented with ketoacid partially decreased the serum albumin loss compared to NPD and LPD groups (compared with NPD, *P* < 0.001; compared with LPD, *P* = 0.001) (Figure 1(e)).

### 3.2. TGF-*β*_1_ and the Receptor

**Figure 2:**
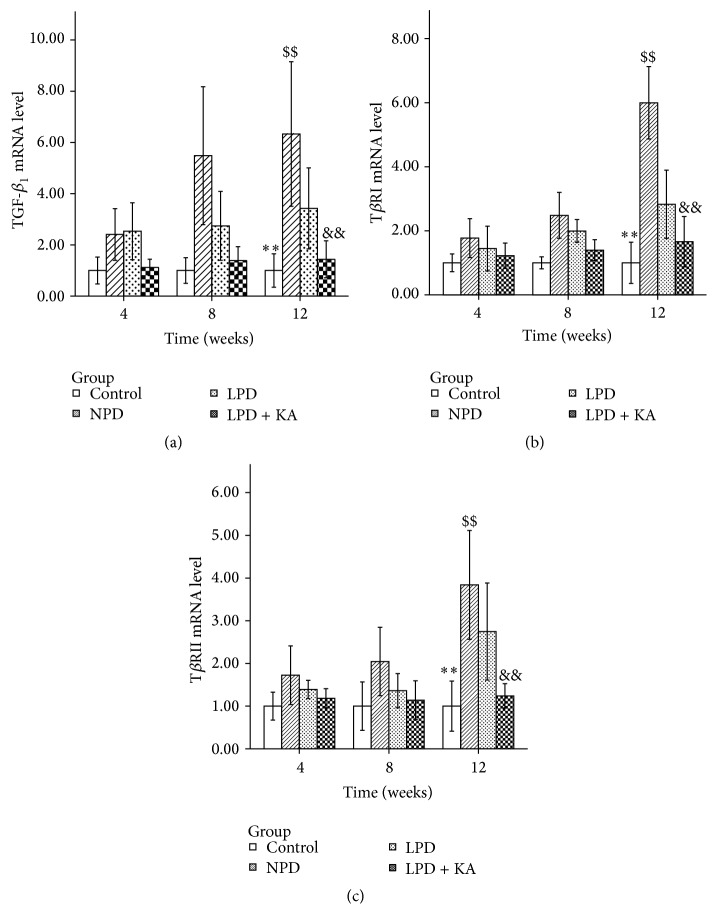
mRNA expressions of TGF-*β* and its receptors in the experimental groups. TGF-*β*_1_ (a), T*β*RI (b), and T*β*RII (c) mRNA levels in the kidney of the experimental groups. Data are expressed as mean ± SD; ^*∗∗*^ *P* < 0.05 versus NPD, LPD, and LPD + KA; ^$$^ *P* < 0.01 versus LPD and LPD + KA; ^&&^ *P* < 0.01 versus LPD. NPD: normal protein diet; LPD: low-protein diet; LPD + KA: low-protein diet supplemented with ketoacids.

Compared to the control group, NPD group exhibited a significant increase of mRNA expression in the related genes TGF-*β*_1_ (Figure 2(a)), T*β*RI (Figure 2(b)), and T*β*RII (Figure 2(c)) (*P* < 0.001). As expected, the low-protein diet with or without addition of ketoacid reduced the increased expression of all these parameters (*P* < 0.001).

Furthermore, ketoacid supplementation significantly reduced the upregulation of these genes. The mRNA expression levels of TGF-*β*_1_, T*β*RI, and T*β*RII in LPD + KA group were significantly lower than those in LPD group (*P* = 0.001, *P* < 0.001, and *P* < 0.001, resp.).

Compared to the control group, all these parameters were significantly higher in LPD group (*P* < 0.001). The mRNA expression levels of TGF-*β*_1_ and T*β*RI in LPD + KA group were insignificantly higher than those in the control group. In addition, the mRNA expression of T*β*RII in LPD + KA group was significantly higher than that in the control group (*P* = 0.029).

**Figure 3:**
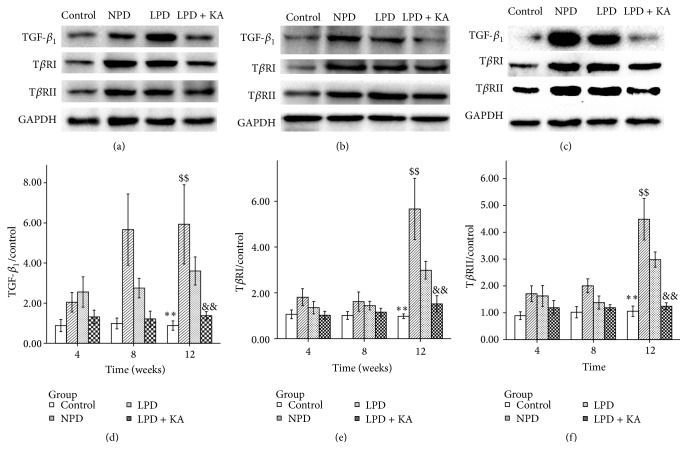
Protein expressions of TGF-*β* and its receptors in the experimental groups. Representative western blotting analyses (a, b, and c) and group data of the abundance of TGF-*β*_1_ (d), T*β*RI (e), and T*β*RII (f) in the kidney of the experimental groups. Data are expressed as mean ± SD; ^*∗∗*^ *P* < 0.05 versus NPD, LPD, and LPD + KA; ^$$^ *P* < 0.01 versus LPD and LPD + KA; ^&&^ *P* < 0.01 versus LPD. NPD: normal protein diet; LPD: low-protein diet; LPD + KA: low-protein diet supplemented with ketoacids.

Similarly, diabetic rats fed with NPD showed a significantly higher level of protein expression in TGF-*β*_1_ (Figure 3(a)), T*β*RI (Figure 3(b)), and T*β*RII (Figure 3(c)) compared to normal rats. In addition, the low-protein diet also caused a decrease in the expression of these proteins, and KA + LPD prevented the overexpression of these proteins more effectively.

## 4. Discussion

Protein restriction has long been recommended as a means to alleviate uremic symptoms, protect the function of remnant kidneys, and improve complications such as abnormal glucose metabolism and hypertension in patients with chronic kidney disease [1, 11, 21].

These metabolic effects resulted in less frequent prescriptions of phosphate binders, allopurinol, bicarbonate supplements [22], and erythropoietin [23]. In studies on diabetic nephropathy, several reports have demonstrated that low-protein diets prevented the progression of renal injury in both humans and animals with DN [24].

Studies [25] have also demonstrated that protein restriction protects against hemodynamic changes in hyperfiltration and progressive sclerosis of functional glomeruli and decreases nitrogen wastes and oxidative stress. Other studies [26, 27] have reported that LPD could slightly slow down the progression of renal failure. Consistent with these findings, we found that LPD decreased SCr, BUN, and urinary protein levels.

Oral nutrient supplements represented by ketoacids have been increasingly favored in recent years, knowing that ketoacids can capture excessive nitrogen residues and utilize them to generate essential amino acids. Therefore, restricting nitrogen intake may reduce the formation of endogenous urea, and ingestion of sufficient amounts of nitrogen-free analogues of essential amino acids seems to reduce the accumulation of nonexcreted, potentially toxic ions and metabolic products arising from the breakdown of foods rich in protein [28].

It was found in our study that the growth of the diabetic rats was retarded and the weight was reduced as compared with the normal rats, suggesting the possibility of insufficient protein synthesis and malnutrition in the diabetic rats. Addition of ketoacids increased the weight of LPD rats and corrected the plasma protein level, indicating that LPD supplemented with ketoacids could improve the nutritional status. LDP + KA preserved all the beneficial effects of LPD on DN and offered an additional protective effect on the kidney in the diabetic rats, confirming the necessity of KA supplementation to LDP.

Numerous experimental and clinical studies have demonstrated that TGF-*β* plays a key role in the pathophysiology of diabetic kidney diseases [29]. It was found that tubular and glomerular expressions of TGF-*β* were increased in patients with both DM1 and DM2 in early and late stages of the disease, and these increased expressions were associated with the degree of glycemic control in these patients [29]. TGF-*β*_1_ was reported to be stimulated by hyperglycaemia and glomerular stretch in the early stages of diabetic kidney disease, and persistent production of TGF-*β* in later stages may be due to stimulation by glycated proteins (e.g., AGEs), the influence of growth factors (angiotensin II (Ang II) and platelet-derived growth factor (PDGF)), and TGF-*β* autoinduction [29]. Ang II has been shown to stimulate the expression of TGF-*β* and its receptors [30, 31].

Multiple mechanisms, including decrease in nitrogen waste, metabolic burden, and oxidative stress, have been suggested for the beneficial effects of LPD supplemented with ketoacid on the progression of CKD. Our finding that chronic inflammation, characterized by increased mRNA expression of TGF-*β*_1_, was substantially reduced in diabetic rats fed with the low-protein or low-protein plus KA diets further supports an antidiabetic-nephropathic action of dietary protein restriction.

The improvement of DN by dietary protein restriction seems to be associated with partial inhibition of TGF-*β*_1_ expression in the kidney, probably by decreasing the oxidative stress and suppressing the increase in the expression of TGF-*β*_1_ and its receptors in the kidney of the diabetic rats fed with low-protein diet and low-protein diet supplemented with KA. In our study, the supplementation of ketoacid largely prevented insufficient protein synthesis or malnutrition in the diabetic rats and abolished the expression of TGF-*β*_1_ and the receptors compared to a sole PLD.

In summary, our study has demonstrated that the levels of TGF-*β*_1_, T*β*RI, and T*β*RII were increased in the renal tissue in DN rats. In addition, a low-protein diet supplemented with ketoacids decreased the expression of TGF-*β*_1_, T*β*RI, and T*β*RII in the renal tissue of DN rats. These findings may provide relevant preclinical data for the use of low-protein diets supplemented with ketoacids in DN patients.
